# Osteoporosis treatments for intervertebral disc degeneration and back pain: a perspective

**DOI:** 10.1093/jbmrpl/ziae048

**Published:** 2024-04-18

**Authors:** Neharika Bhadouria, Nilsson Holguin

**Affiliations:** Department of Orthopedics, Icahn School of Medicine at Mount Sinai, New York, NY 10029, United States; School of Mechanical Engineering, Purdue University, West Lafayette, IN 47907, United States; Department of Orthopedics, Icahn School of Medicine at Mount Sinai, New York, NY 10029, United States

**Keywords:** animal models, anabolics, antiresorptives, hormone replacement/receptor modulators, nucleus pulposus, osteoblasts, osteocytes, osteoclasts

## Abstract

Low back pain derived from intervertebral disc (IVD) degeneration is a debilitating spinal condition that, despite its prevalence, does not have any intermediary guidelines for pharmacological treatment between palliative care and invasive surgery. The development of treatments for the IVD is complicated by the variety of resident cell types needed to maintain the regionally distinct structural properties of the IVD that permit the safe, complex motions of the spine. Osteoporosis of the spine increases the risk of vertebral bone fracture that can increase the incidence of back pain. Fortunately, there are a variety of pharmacological treatments for osteoporosis that target osteoblasts, osteoclasts and/or osteocytes to build bone and prevent vertebral fracture. Of particular note, clinical and preclinical studies suggest that commonly prescribed osteoporosis drugs like bisphosphonates, intermittent parathyroid hormone, anti-sclerostin antibody, selective estrogen receptor modulators and anti-receptor activator of nuclear factor-kappa B ligand inhibitor denosumab may also relieve back pain. Here, we cite clinical and preclinical studies and include unpublished data to support the argument that a subset of these therapeutics for osteoporosis may alleviate low back pain by also targeting the IVD.

## Graphical Abstract

**Graphical Abstract:**
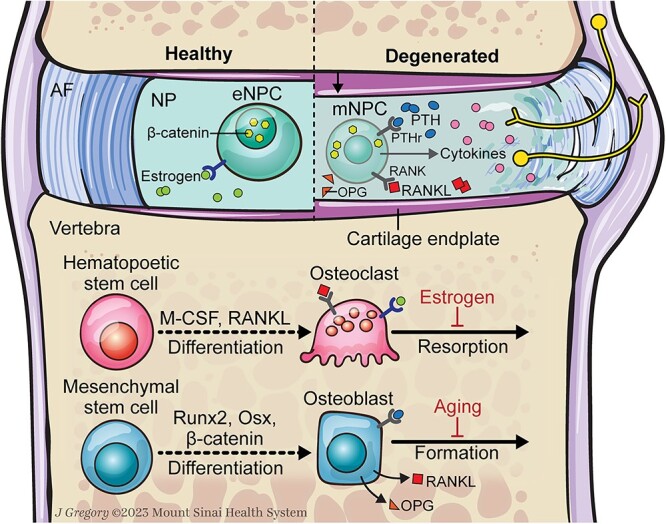

## Introduction

*Motivation.* Intervertebral disc (IVD) degeneration is a major etiological factor of low back pain, which carries an estimated economic burden of at least $100 billion in treatment to United States Americans and is the #1 cause of job disability worldwide.^1^^,^^2^ The study of IVD degeneration is a very needful field as IVD degeneration is implicated in accounting for ~40% of back pain patients.^3^^,^^4^ (1) Aging is a common risk factor for IVD degeneration^5^ and low back pain,^6^^,^^7^ and by the 2030s, the US geriatric population is projected to outnumber children for the first time in history.^8^ (2) Treatment for IVD degeneration is either palliative or highly invasive, with limited intermediate options for treatment.^1^^,^^2^ There is a sensitive homeostatic relationship between vertebral bone and IVD, where disruptions in bone mass can impact the health of the IVD. Both low^9^ and high^10^ bone mineral density (BMD) of lumbar vertebrae are associated with IVD degeneration; where cartilage endplate thinning could disrupt the normal transmission of mechanical forces to the IVD and cartilage endplate calcification could disrupt solute transport to the IVD, respectively. While current pharmacological therapies for osteoporosis all improve bone structure and may consequently improve IVD structure,^10^ we propose that particular osteoporosis drugs may also relieve back pain via IVD-related mechanisms independent of bone.

*IVD Composition and Degeneration.* Many studies have described the progression of IVD degeneration.^5^^,^^11^ In a healthy IVD, the central nucleus pulposus (NP) of the IVD absorbs shock and transfers forces radially to the annulus fibrosus (AF) of the IVD, where the forces are converted to circumferential stress. The thin layer of cartilage endplate (CEP) between the vertebra and the IVD transmits forces to the IVD, pressurizes the NP and permits the transport of water, solutes, nutrients and waste. The cells of the IVD are categorized by the region in which they reside, where NP cells are derived from notochordal cells and AF cells are sclerotome-derived. CEP cells are chondrocytic and also derived from the sclerotome.^12^^,^^13^In late-stage IVD degeneration, IVDs are severely dehydrated, thinner in height, solid-like and biomechanically stiffer.^14^^,^^15^ IVD dehydration is linked to a concentrated loss of glycosaminoglycans in the nucleus pulposus,^15^ which may lead to the development of fissures in the outer annulus fibrosus and posterior IVD protrusions.^5^^,^^16^^,^^17^ Complete IVD collapse leads to bone-to-bone friction, which can instigate inflammation and painful sensations. Aging can exacerbate features of IVD degeneration, e.g., structural disorganization, dehydration, extracellular matrix breakdown, height loss, apoptosis, among other features.^18–21^

*Back Pain and Osteoporosis.* IVD degeneration is a major contributing factor of low back pain, which is most notably associated with inflammation^3^ and nerve recruitment by the expression of tachykinin neuropeptide Substance P (Graphical Abstract).^22^ Pre- and postmenopausal women develop more severe back pain than age-matched men, respectively, and postmenopausal women are more susceptible to IVD degeneration than men.^1^^,^^23^ Women experience pain more frequently and at higher intensities than men^24^^,^^25^ and have a higher propensity for osteoporosis and a greater incidence of skeletal fracture than men, which underscores the overall and sex-specific need for the development of treatment.

It is unsurprising that skeletal fracture is highly associated with back pain,^26^ but possibly more surprising are the numerous reports of treatments for osteoporosis that alleviate back pain without prior skeletal fracture. Briefly, bone structure and maintenance is regulated by three major cell types: bone-building osteoblasts, bone-resorbing osteoclasts and bone-orchestrating osteocytes. This perspective will focus on common therapies that target osteoblasts and osteoclasts to reduce the risk of skeletal fracture, but may also alleviate back pain via discogenic mechanisms, and are therefore neither intended to compare treatment efficacy^27^ nor to decide treatment options.^28^

*Bisphosphonates and bone-centric reduction of back pain.* The most notable exclusion in this perspective will be of bisphosphates (BPs) because the mechanism by which they function to alleviate back pain appears to be strictly bone-dependent. BPs are the most commonly used treatments for the prevention of osteoporotic fracture in postmenopausal women owing to their high binding affinity to hydroxyapatite crystals in bone and release at sites of active remodeling to promote bone accrual.^29^^,^^30^ Bisphosphonates are derivatives of inorganic pyrophosphate and the first-generation BPs of etidronate, clodronate and tiludronate included a hydroxyl group to bind to calcium and a phosphate group to increase affinity to hydroxyapatite crystals.^31^ Next-generation BPs alendronate, risedronte, pamidronate, and zolendronic acid include nitrogen side chains promote osteoclast apoptosis and induce orders of magnitude greater potency than the first-generation iterations.^29^ BP-use in humans with a pre-existing vertebral condition, e.g., modic change or fracture,^32–34^ and in ovariectomized mice^35^^,^^36^ reduce indications of low back pain and its markers. The mechanism by which BPs reduce back pain is unlikely to be IVD-centric. Although aging and degeneration may promote IVD calcification,^37^^,^^38^ serving as scaffold for BPs, IVDs do not have osteoclasts to resorb and release BPs. Secondly, ovariectomy does not overtly calcify the IVD^39^^,^^40^ and the quick benefit of BPs like alendronate to spontaneous and evoked pain measures of ovariectomized mice is unlikely due to resorption of calcification in the IVD.^35^

## Objectives

Unsurprisingly, the cells of the bone and IVD share molecular pathways critical for cell maturity and tissue homeostasis that are currently therapeutic targets to maintain bone structure and prevent skeletal fractures. We have two goals with this perspective: (1) JBMR Plus’ open access policy will help promote the uncommon knowledge that some osteoporosis treatments may reduce back pain via mechanisms separate from the attenuation of vertebral fracture risk; and (2) we are calling for further investigation of the understudied effects and mechanisms underlying back pain relief by the administration of widely used osteoporosis treatments.

## Materials and methods

*Search Criteria*. The literature search keywords and criteria for each drug category in pubmed can be categorized by the text sections, which are “Mechanism(s) of Action in Bone,” “Effects on Back pain and (IVD) Height,” and “Putative Mechanism(s) of Action in IVD. For “Mechanism(s) of Action in Bone,” a particular focus is placed on the clinical trials for keywords ‘bone’ and the respective drug: ‘parathyroid hormone’ (PTH) or ‘teriparatide’, ‘anti-sclerostin antibody’ or ‘romosozumab,’ ‘raloxifene,’ or ‘denosumab’ from 1970 to 2024. For “Effects on Back Pain and (IVD) Height,” the additional search criteria include the keywords ‘low back pain,’ ‘back pain’ or ‘pain.’ For “Putative Mechanism(s) of Action in IVD,” keywords for the respective drug are additionally combined with the keywords ‘intervertebral disc,’ ‘nucleus pulposus,’ or ‘annulus fibrosus.’ Search results for each section are mutually inclusive for references regarding the osteoporosis drug.

*Acute Versus Chronic Back Pain*. Acute pain may occur in response to an injury, whereas chronic pain is defined as pain in 1 or more sites lasting 3 months or more.^41^ The distinction between acute (new) back pain and chronic back pain in this perspective will be made if the reported study indicates the continuity and/or duration of pain. If the history of patient-reported back pain in the cited study is not listed, then the “back pain” will be listed as acute. For the studies using animal models, the terms “pain-like” or “pain-related” behavior will be used in reference to pain, unless the duration of pain is measured as lasting longer than 3 months.

## Results - Bone formation

### Intermittent parathyroid hormone - Teriparatide

*Mechanism(s) of Action in Bone.* Intermittent PTH injections, or the analogous teriparatide that is composed of the first 34 amino acids (1—34), stimulate bone formation and inhibit bone resorption.^42^^,^^43^ While low and short-term dosing of intermittent PTH is osteoanabolic,^44^^,^^45^ high dosing stimulates bone resorption and induces hypercalcemia.^45^ Whole PTH is composed of 84 amino acids produced by parathyroid gland cells and can maintain serum calcium by directly stimulating the PTH receptors on osteoblasts and osteocytes to activate adenylyl cyclase/cyclic AMP (cAMP)/protein kinase A (PKA) signaling.^46^ Indirectly, excessive PTH stimulates osteoblastic lineage cells to promote the ratio of receptor activator of nuclear factor-kappa B ligand (RANKL) to its soluble decoy receptor osteoprotegerin (OPG) and, in turn, stimulates osteoclasts to resorb bone (Graphical Abstract).^47^^,^^48^

The Fracture Prevention Trial is a landmark study that established that the benefits of short-term injections of PTH on bone structure and fracture risk can persist long-term.^44^ A total of 1637 postmenopausal women with prior history of fracture were assigned to once daily injection of 20 or 40 μg of 1–34 or placebo for 2 weeks. Injection of 1–34 at 20 and 40 μg reduced the relative risk of a new vertebral fracture by 64–71% for up to 24 months (20 μg, relative risk of 0.35, 95% CI of 0.22 to 0.55, 40 μg, relative risk of 0.31, 95% C) of 0.19 to 0.50). Injection of 1-34 induced similar benefits to non-vertebral fracture risk and bone mineral density.

*Effects on Back Pain*. The Fracture Prevention Trial^44^ and subsequent studies^49^ demonstrate that teriparatide reduces the incidence of new or worsening back pain and promotes stature by reducing vertebral fractures in postmenopausal women (Table 1). Back pain in these studies was defined as impairment of physical activity and need of pain relief, and was not defined as chronic pain. Twenty-three percent of women on placebo reported new or worsening back but injection of teriparatide reduced this incidence by 5–6%. Corroboratively, women who experienced one or more new vertebral fractures lost less height on teriparatide (20 and 40 μg) than placebo (−0.2 cm, −0.3 cm vs −1.1 cm). A secondary analysis of the Fracture Prevention Trial data in a follow-up study^49^ further stratified the benefit of teriparatide at 20 μg to relieve back pain in postmenopausal women with a previous fracture. The relative risk of developing moderate or severe back pain is reduced by 31% in teriparatide-treated compared to placebo-treated patients.

**Table 1:** Type of low back pain assessed in clinical studies using anti-osteoporosis pharmacological treatment.

Type of Pain	Teriparatide	Romosozumab	Raloxifene	Denosumab
Acute	Neer et al.,^44^	Cosman et al.^69^	-	Takeuchi et al.^97^
	Genant et al.,^49^			
	Miller et al.,^51^			
	Aloumanis et al.^53^			
Chronic	Lyritis et al.,^50^	-	Lyritis et al.,^50^	Cai et al.^34^
	Hadji et al.,^52^		Fujita et al.^85^	
	Aloumanis et al.^53^			

The benefits of teriparatide on acute and chronic back pain relief occur within the first year.^50–54^ These data from the Fracture Prevention Trial^49^ estimated that the benefits to back pain relief over the course of the 2-year study may have begun at 12 months. A 2-year study (EUROFORS - EUROpean study of FORSteo) that enrolled 868 postmenopausal women with osteoporosis and a recent fragility fracture^50^ monitored the incidence of back pain after 12 and 24 months of teriparatide. After 12 months of teriparatide (20 μg/day), a subset of patients (n = 507) were randomized to continue teriparatide (*n* = 305), raloxifene (60 mg/day, *n* = 100), or no treatment (*n* = 102) for another 12 months. Patients self-assessed back pain using a visual analogue scale (0–100 mm) and, after the initial reduction in self-assessed back pain by 12 months of teriparatide, 12 additional months of teriparatide did not statistically change the incidence of back pain (the raloxifene-related results are discussed in a next section). The European Forsteo Observational Study suggests that teriparatide injection may even reduce the incidence back pain as early as after 3 months of treatment.^53^

Vertebral fractures increase the incidence and severity of back pain and, although several bone therapeutics reduce the risk of vertebral fracture, comparison studies show that teriparatide is uniquely effective at reducing back pain in patients on the precipice of experiencing a new vertebral fracture. Compared to bisphosphonates alendronate^50^ and risedronate,^52^ which both reduce the incidence of vertebral fracture, teriparatide more greatly reduces the intensity and number of patients with back pain.^49^ More specifically, compared to placebo treatment, injection of 20 μg of teriparatide reduces the incidence of back pain in patients with 1 or more of fractures and those with moderate or severe fracture.^49^ However, the incidence of new or worsening back pain, may it be mild, moderate or severe, was not reduced by teriparatide treatment in patients without a new vertebral fracture. Similarly, in the EUROFORS study, teriparatide injection reduced the incidence of back pain irrespective of one or more vertebral fractures, but teriparatide injection induced a greater reduction of back pain in those with a recent fracture.^50^ These studies suggest two conclusions about the resolution of back pain by teriparatide injections: (i) bone accrual by the stimulation of osteoblasts and osteoclasts mitigates fracture risk and subsequent back pain and (2) the reduction of fracture incidence alone is insufficient to reduce back pain, as noted by the lack resolution of back pain by injection of teriparatide in patients without a vertebral fracture. Overall, these studies suggest that PTH-induced reduction in back pain may primarily involve stimulation of bone cells and secondarily involve another cell type.

*Putative Mechanism(s) of Action in IVD*. Much of the preclinical literature on the efficacy of PTH to limit low back pain rely on a baseline bone phenotype, where the bone can be at the precipice of vertebral fracture^49^^,^^55^ or had recently experienced a fragility fracture.^50^ Consequently, animal studies have administered PTH in the context of spinal challenges that impair vertebral bone structure to find that, concomitant to greater vertebral bone structure, PTH can improve IVD composition and limit pain-like behavior. For instance, subcutaneous injections of teriparatide to mice after 6 weeks of ovariectomy, metrics indicative of pain-like behavior, e.g., von Frey, paw-flick test and spontaneous evoked acute pain after 3 days, return to levels similar to ovariectomy upon discontinuation.^35^ Therefore, resolution of back pain following resolution of a challenged baseline bone phenotype may not necessarily involve the IVD.

However, there may be a bone-independent, IVD-direct mechanism by which PTH may function to limit the development of discogenic pain. Human IVD cells express PTH 1 receptor (PTH1R)^56^^,^^57^ and PTH can stimulate human annulus fibrosi and nucleus pulposi to upregulate extracellular matrix gene and protein expression. Similarly, intermittent PTH in mice promotes IVD height and reduces features of IVD degeneration in aged mice by activating transforming growth factor-β through primary cilia (Table 2).^58^ By contrast, deletion of PTH1R induces extracellular matrix breakdown, weakens IVD flexibility and aggravates mechanically-induced IVD degeneration.^58^ Tail suspension is an animal model to induce mild IVD degeneration^59–61^ and subcutaneous injection of teriparatide may partially normalize the suspension-induced dysregulation of gene and protein expression for extracellular matrix, cytokines and apoptosis markers in the IVD.^56^ Similarly, in vitro exogenous PTH on human nucleus pulposus cells promotes cell viability and reduces the expression of ColX in human nucleus pulposus cells,^56^^,^^57^ which is associated with aging-related maturation of nucleus pulposus cells.^20^

**Table 2:** Effect of bone therapeutic and genetic manipulation/reduction of target to in vivo rodent spine.

Outcome	iPTH^58^^,^^62^	PTH1R KO^58^^,^^62^	Scl-Ab^73^	β-Catenin cACT^75^ / SOST KO^73^	β-Catenin^75^ / LRP5^74^ KO	Ralox^38^	OVX^39^^,^^40^^,^^109^	Dmab^40,^*	OPG KO^101^^,^^102^
Vertebral Bone	↑	↓	↑	↑	↓	↑	↓	↑	↓
IVD Degeneration	↓	↑	NC	NC	↑	↓	↑	↓	↑
IVD Height	↑	NC/↓	↑	↑	↓	↑	↓	↑	↓
IVD Mechanics	↑	↓	↑	↑	↓	↑	↓	-	-

^*^: Denosumab (Dmab) was injected during spinal challenge and not in naïve animals. “-”: not measured, “NC” – no change, “Ralox” – raloxifene.

By contrast, conditional deletion of PTH1R in nucleus pulposus cells using the Noto-cre driver does not impair the improvement in pain-like behavior by administration of PTH.^62^ Further, PTH reduced endplate innervation in advanced aging and mechanically-induced IVD degeneration and improved pain-related behavior. One potential caveat of this excellent study is that annulus fibrosus cells, which also express PTH1R, were not targeted and may still have contributed to the PTH-related improvements. Therefore, PTH also may serve to promote or maintain immature nucleus pulposus cells in the IVD, which are better equipped to produce extracellular matrix, or annulus fibrosus cells in mitigating back pain.^62^^,^^63^

### Anti-Sclerostin antibody - Romosozumab

*Mechanism(s) of Action in Bone.* FDA-approved romosozumab (brand name Evenity by Amgen and UCB) is an anti-sclerostin-antibody that systemically allows the activation of Wnt/β-catenin signaling in cells that express sclerostin and may influence the IVD.^64–68^ Sclerostin is an inhibitor of the Wnt/β-catenin signaling pathway and global suppression of sclerostin by systemic injection of Scl-Ab or genetic ablation of its precursor *SOST(human)/sost(mouse)* promotes bone formation in osteoblasts and mildly limits bone resorption by osteoclasts (Graphical Abstract).^5^

A total of 7168 osteoporotic postmenospausal women were enrolled in a randomized, double-blind, placebo-controlled, parallel-group trial to receive placebo or 210 mg of monthly SQ of romosozumab for 12 months and, subsequently, both groups received 60 mg of denosumab every 6 months for an additional 12 months.^69^ Romosozumab injection reduced the relative risk of a new vertebral fracture by 73–75% (12 months: relative risk of 0.27, 95% confidence interval (CI) of 0.16–0.47; 24 months: relative risk of 0.25, 95% CI of 0.16–0.40). At 12 months, romosozumab injection increased bone mineral density at the lumbar spine by 13% (CI: 11.9–14.7), which coincided with greater serum expression of bone formation marker P1NP and less serum expression of β-CTX. The most common adverse event from romosozumab injection was injection-site pain in 1.7% of the patients.

*Effects on Back Pain*. Over the course of 12 months of injections, patients in the placebo group (378/3576, 10.6%) and romosozumab group (375/3581, 10.5%) reported a similar incidence of acute back pain.^69^ Although injection of romosozumab did not affect the incidence of back pain, the incidence of back pain was an order of magnitude greater than the incidence of new vertebral fracture. Therefore, vertebral fracture is not the only mechanism driving back pain in these patients. Further, mutation of *SOST* in sclerosteosis patients is associated with greater bone mass and body stature.^70^ Similarly, injection of scl-Ab in mice,^71^ bone cell-conditional deletion of *sost*, and global KO of *sost* increase bone mass and do not affect vertebral bone length.^72^ Therefore, sclerosteosis patients may have greater stature and spinal elongation from greater IVD height.

*Putative Mechanism(s) of Action in IVD*. *Sost* and its Wnt signaling receptor LRP5 are expressed in the entire IVD,^73^^,^^74^ and neutralization of sclerostin may allow activativation of Wnt signaling in human IVD. Global deletion of *sost* in 4-month-old mice increases extracellular matrix anabolism, IVD height and hydration as determined by MRI (Table 2). Conditional stabilization of β-catenin in sonic hedgehog-expressing (SHH) cells induce similar IVD benefits to global sost KO and strengthen IVD mechanical properties.^75^ Similarly, injection of 25 mg/kg of anti-sclerostin antibody, 25 mg/kg of anti-DKK1 antibody (another Wnt signaling inhibitor) or 3:1 combinatorial injection with anti-sclerostin:anti-Dkk1 antibody (18.75,6.25 mg/kg), to 9 wk old female C57Bl/6 mice all upregulated *β-catenin* gene expression and increased IVD height.^73^ Uniquely, injection of anti-sclerostin antibody strengthened the mechanical stiffness of the IVD but none of the other dosage combinations improved the mechanical properties of the IVD. These data suggest that the mechanism by which neutralization of sclerostin affects the IVD is not limited to canonical Wnt signaling via transcription factor β-catenin. For example, Wnt signaling inhibitor DKK1 is a target of Wnt signaling^76^ and neutralization of Dkk1 does not regulate heat shock proteins (HSPs) gene expression in the IVD. By contrast, although Wnt signaling is normalized in sost KO IVD by upregulation of Dkk1 and other Wnt signaling inhibitors, both global sost KO and neutralization sclerostin downregulate HSPs. Hsps mediate axial patterning and cell proliferation, and long-term induction of Hsps reduces Wnt targets brachyury and TCF.^77^

Lastly, the functional, structural and cellular benefits of anti-sclerostin antibody to murine IVD do not appear to translate to reduced incidence of back pain in postmenopausal women.^69^ Estrogen and Wnt signaling interact and potentiate each other upon stimulation.^78^ In the IVD, injection of estrogen agonist raloxifene upregulates *β-catenin* gene expression and the number of β-catenin-positive cells in the IVD.^39^ Therefore, estrogen-deficiency in postmenopausal women may limit the benefit of romosozumab on discogenic back pain. In addition, bone formation requires innervation^79^ and the accumulation of osteocytes expressing neurotransmitter substance P (SP) in vertebral bone by anti-sclerostin antibody^73^ may counter balance the other discal benefits.

## Bone anti-resorptives

### Selective estrogen receptor modulator (SERM) - Raloxifene

*Mechanism(s) of Action in Bone.* Raloxifene hydrochloride is an FDA-approved therapeutic that reduces fracture-risk in postmenopausal women. Raloxifene reduces fracture risk by at least two mechanisms: by (i) cell-dependent and (2) cell-independent mechanisms. The cell-dependent mechanism is the canonical understanding, where raloxifene binds to the estrogen receptors (ER) in osteoclasts to suppress bone resorption (Graphical Abstract).^80^^,^^81^ In the Multiple Outcomes of Raloxifene Evaluation study,^82^ the spinal radiographs of 7705 women aged 31 to 80 years who were postmenopausal for at least 2 years were evaluated to determine vertebral fracture incidence at baseline and after 24 months and 36 months of treatment. Dual-energy x-ray absorptiometry was used to determine bone density. Participants were randomized to receive placebo pills, 60 or 120 mg/d. Both dosages of raloxifene reduced the relative risk (RR) of vertebral fracture (60 mg/d: RR of 0.7, 95% confidence interval (CI) of 0.5-0.8; 120 mg/d: RR of 0.5, 95% CI of 0.4-0.7). Corroboratively and compared to placebo, raloxifene increased vertebral bone mineral density by 2.6% and 2.7% in the 60 or 120 mg/d groups, respectively. However, the mild 3% increase in bone mineral density does not fully explain the 50% reduction of vertebral fracture risk. The second mechanism by which raloxifene may reduce the relative risk of vertebral fracture is by elevated hydration of bone. Independent of ER and living cells, raloxifene can chaperone water to the surface of collagen, and consequently can increase the mechanical toughness of bone without increasing mineral content.^83^^,^^84^

*Effects on Back Pain*. Estrogen agonist raloxifene reduces self-assessed chronic back^50^ and joint pain,^85^ and IVD height in women is associated with estrogen status. A prospective, controlled, randomized, open-label, 2-year study enrolled 868 postmenopausal women with osteoporosis and a recent fragility fracture.^50^ After 12 months of teriparatide injections (20 μg/day), a subset of patients (n = 507) were randomized to continue teriparatide (*n* = 305), raloxifene (60 mg/day, *n* = 100), or no treatment (*n* = 102) for another 12 months. Patients self-assessed back pain using a visual analogue scale (0–100 mm). After the initial reduction in self-assessed back pain by 12 months of teriparatide injections, 12 additional months of teriparatide injections did not statistically change back pain, but switching to 12 months of raloxifene treatment after 12 months of teriparatide injections further reduced self-assessed back pain. Similarly, menopausal women have less IVD height than premenopausal and, compared to nontreated postmenopausal women, hormone replacement therapy of estrogen increases the IVD height of postmenopausal women.^86^

*Putative Mechanism(s) of Action in IVD*. Administration of raloxifene may be beneficial to the IVD by a number of mechanisms. Mechanism 1) Cell-independently, raloxifene may increase IVD height by binding greater water to collagen. Mechanism 2) Cell-dependently, raloxifene and estrogen also bind to ERα and β^87^ and promote the proliferation of nucleus pulposus and annulus fibrosus cells (Table 2).^88^ These cellular triggers by estrogen agonism may explain the stimulation of Wnt signaling and consequent attenuation of ovariectomy-induced IVD degeneration in rats,^89^ supporting the rationale for the use of raloxifene in women, which express fewer ER than men in the IVD^90^ and are at greater risk of developing IVD degeneration.^91^ Mechanism 3) Neurokinin-1 (Substance P), a neurotransmitter and nociceptive pain marker, is negatively associated with ERα/β protein expression in the IVD of elderly women.^90^ Overall, these bone prophylactics can preserve the IVD, prevent IVD degeneration, and show particular promise for the elderly, who are in great need of protection.

Studies in mice suggest that injection of raloxifene can improve IVD properties independent of bone. Estrogen replacement therapy of 17β-estrogen at a dose of 25 μg/kg for 5 days per week for 8 weeks partially protects from ovariectomy-induced IVD degeneration in rats.^89^ Partial recovery in the IVD includes benefits to the histological IVD degeneration score, extracellular matrix gene and protein expression, and mechanical properties of the spinal motion segment. However, these studies are based on the challenge induced by OVX on the IVD and, therefore, the benefit of the estrogen therapy on the IVD could be dependent on the neighboring vertebra. Similarly, injection of estrogen agonist raloxifene at a dose of 0.5 mg/kg for 5 days per week for 6 weeks to young-adult and old female mice increases lumbar vertebral bone structure, reduces the histological grading score IVD degeneration, promotes extracellular matrix gene expression and mechanically strengthens the IVD.^39^ Dissimilarly, injection of raloxifene to male mice did not alter lumbar vertebral bone structure, did not upregulate estrogen receptor alpha protein expression in the IVD, nor did it improve mechanical properties of the IVD. Although biological sex limits bone accrual and ER alpha upregulation in the IVD by raloxifene in male spines, injection of raloxifene in male mice increases IVD height, promotes Wnt/β-catenin signaling, and reduces gene and protein expression of pain-neurotransmitter *tac1*/substance P. These data suggest that the benefits of raloxifene to the IVD may occur without stimulation of bone cells and may also be independent of estrogen signaling.

**Figure 1:**
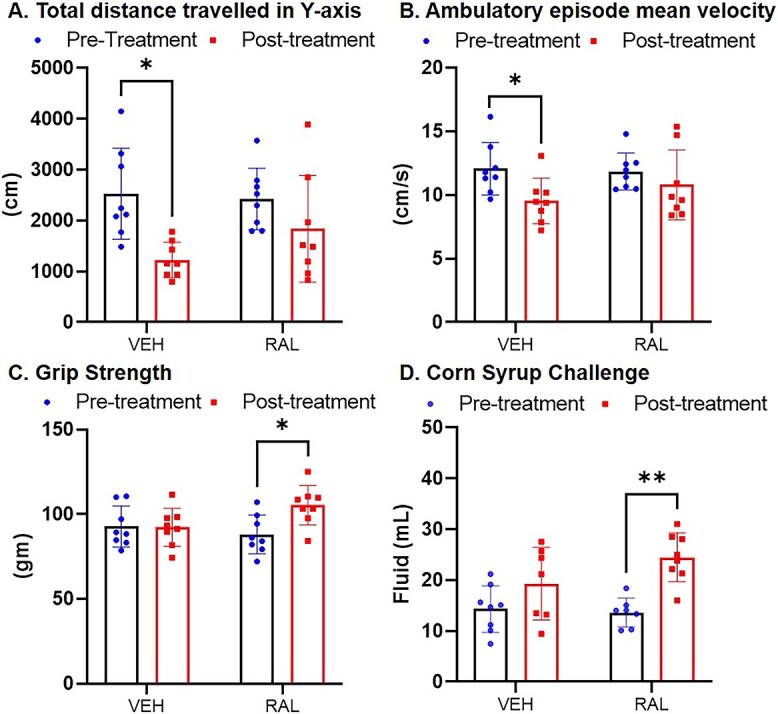
Injection of raloxifene to 22.5-month-old female mice for 6 weeks maintained (A) the distance traveled in the y-axis and (B) ambulatory episode mean velocity and increased (C) 4-paw grip strength and (D) intake of corn syrup. *P < 0.05, ** *P* < 0.01.

Further, injection of raloxifene can improve the physical metrics of pain-related behavior in old female mice. Under IACUC approval, 22.5 month old C57Bl/6 female mice were monitored over 6 weeks of daily treatment with vehicle PBS or raloxifene (n = 8/grp, 0.5 mg/kg). At baseline/pre-treatment in an open field test, the mice traveled the same distance and their peak-velocity over a two-hour window at night was the same between vehicle-treated (VEH, PBS) and raloxifene-treated (RAL, Figure 1). After 6 weeks, vehicle-treated old female mice did not maintain the rate of distance traveled and ambulatory episode mean velocity at baseline (*P* < 0.001, Figure 1A and B). By contrast, 6 weeks of injection of raloxifene maintained the rate of distance traveled, maintained the ambulatory episode mean velocity, and increased the grip strength of old female mice, Figure 1A-C). Therefore, at baseline and day 28, we tested the preference for corn syrup over water, which is an indicator for pleasure-seeking that is hampered by pain. For instance, induction of systemic inflammation by lipopolysaccharide increased Substance P and reduced sucrose intake but injection of an antagonist for substance P improved corn syrup intake.^92^ At baseline and day 28, mice in both groups chose corn syrup over water at similar amounts (*data not shown*). By contrast, corn syrup intake was greater in old mice treated with raloxifene than vehicle PBS (Figure 1D), suggesting that these mice were in less pain and sought greater pleasure. Pain relief in the spine may have promoted physical activity and pleasure-seeking. A substantial body of work suggests that raloxifene can treat brain injuries because of its neuroprotective and anti-inflammatory abilities.^93^

### Denosumab

*Mechanism(s) of Action in Bone.* Receptor activator of nuclear factor-kappa B ligand (RANKL) is a member of the TNF ligand super family that binds to RANK receptors on osteoclast precursors to drive differentiation and activity (Graphical Abstract).^94^ Osteoprotegerin (OPG) is a soluble decoy receptor for RANKL and denosumab is an anti-RANKL inhibitor that inhibits osteoclastogenesis to promote bone accrual.^95^ In brief, the Fracture Reduction Evaluation of Denosumab in Osteoporosis Every 6 months (FREEDOM) was a randomized, multicenter, double-blind, placebo-controlled study that evaluated fracture prevention in postmenopausal women by denosumab.^96^ Subjects received subcutaneous injections of 60 mg of denosumab or placebo every 6 months for 36 months. Denosumab reduced new vertebral fracture incidence by 68% (RR of 0.32; 95% CI, 0.26–0.41),^96^ but also increased the risk osteonecrosis of the jaw.^95^ Promising alternatives include aptamers, which are single-stranded RANKL-targeting oligonucleotides and have less severe adverse effects than denosumab.

*Potentially Relieves Back Pain*. The FREEDOM study did not evaluate back pain but a small, single-center, double-blind, placebo-controlled, randomized parallel-group trial of single dose of 60 mg of denosumab reduces low back pain (LBP), as measured by the “LBP Rating Scale” and the visual analog scale over 6 months, in patients with an history of LBP and modic changes in the bone marrow.^34^ However, the DESIRABLE study tested the effect of 60 mg of denosumab on the progression of bone erosion and BMD in patients with rheumatoid arthritis to show that acute back pain was not different between treatment and placebo.^97^

*Putative Mechanism(s) of Action in IVD.* While the deletion of RANKL clearly induces osteopetrosis (high bone mass)^98^ and deletion of RANKL decoy OPG induces bone loss,^99^ only a couple of studies have investigated the effect of expression and regulation of RANKL/OPG system on the IVD. First, human nucleus pulposus and annulus fibrosus cells express RANK receptor, ligand and decoy OPG, and recombinant human IL-1β and moderately severe IVD degeneration increase their expression.^100^ Images by x-ray and reconstruction of microCT scans suggest that global KO of RANKL increases IVD height, whereas administration of soluble RANKL and RANKL KOs normalize IVD height (Table 2).^98^ In the IVD, global deletion of OPG upregulates the gene expression of inflammatory cytokines, anti-catabolic ECM, chondrogenesis and cell death.^101^^,^^102^ Denosumab is an anti-RANKL antibody and Sun et al.^40^ show that 4 weeks after a single injection of denosumab to 3 month-old rats subjected to ovariectomy and spinal fusion improved vertebral bone structure, bone mineral density and IVD height index, which is the IVD height relative to the length of the bone.^40^ Moreover, denosumab may have reduced the incidence of large cell clusters in the nucleus pulposus, which is a hallmark sign of early IVD degeneration, along with reduction of protein and gene expression markers of chondrogenesis and extracellular matrix breakdown. These data suggest that the RANK/RANKL/OPG system may be involved in IVD chondrogenesis and additional common features of IVD aging.^74^

## Conclusion

Here, we reviewed the literature to support or, when available, refute the argument that osteoporosis drugs may alleviate back pain beyond targeting bone cells by also targeting the IVD. The literature is moderately populated with exciting data to support this argument, but there are a few knowledge gaps that require bridging to clarify the mechanism of action. First, more clinical studies may need to exclude patients with acute (and possibly sporadic) back pain to determine the effect of treatment on chronic back pain. Second, to determine the tissue-specific mechanism by which therapeutics may relieve chronic back pain, the experimental approach may also need to exclude patients with a prior vertebral fracture. In animals studies, administration of anti-osteoporosis drugs should also include naïve animals without a pre-existing spinal challenge, e.g., ovariectomy, that could cloud the tissue-specific mechanism of action. Further, a conditional KO of the bone-related target during treatment would be optimal to determine whether bone cells are absolutely necessary to relieve back pain, as noted in some clinical trials. Next, preclinical spinal pain research should consider the animal model and their subsequent implications cautiously^103^ as features of the spine are not the same across species and should incorporate behavioral phenotyping,^104^ histological IVD scoring,^105–108^ and functional characterization of the IVD. Overall, research incorporating the study of both vertebral bone and the neighboring fibrocartilage of the IVD would help to resolve the underlying etiology of spinal pain and would offer new possibilities from well-studied drugs.

## Data Availability

Data will be made available upon reasonable request.
